# Supplementary material for: Transcriptome analysis of Xanthomonas oryzae pv. oryzicola exposed to H2O2 reveals horizontal gene transfer contributes to its oxidative stress response
Source: PLoS One. 2019 Oct 3;14(10):e0218844. doi: 10.1371/journal.pone.0218844 (PMC6776340; doi:10.1371/journal.pone.0218844)
Supplement: S1 Table — (DOCX) [file pone.0218844.s001.docx]

**S1 Table.** Strains and plasmids used in this study.

| Strains and plasmids | Characteristics | Source |
| --- | --- | --- |
| **Strains** |  |  |
| *Escherichia coli* DH5a | F80 lacZDM15, D(lacZYA-argF)U169, *rec*A1 | Laboratory collection |
| *Escherichia coli* S17-1λ pir | F- *rec*A, hsdR, RP4-2 (Tc::Mu) (Km::Tn7) lysogenized with λpir phage | Laboratory collection |
| BLS256 | wild-type strain of *Xanthomonas oryzae* pv. *oryzicola* | Bogdanove et al. 2011 |
| *Δ1643* | deletion of xoc_1643 in wild-type strain BLS256 | This study |
| *Δ1946* | deletion of xoc_1946 in wild-type strain BLS256 | This study |
| *Δ0582* | deletion of xoc_0582 in wild-type strain BLS256 | This study |
| *Δ2868* | deletion of xoc_2868 in wild-type strain BLS256 | This study |
| *Δ3249* | deletion of xoc_3249 in wild-type strain BLS256 | This study |
| **Plasmids** |  |  |
| pkMS1 | Suicide vector derived from pK18mobGII, sacB ^+^ ; Km ^R^ | Guo et al. 2012 |
| pkMS1-*1643* | Km^R^; pKMS1 containing the left (196 bp) and right (434 bp) DNA fragment of xoc_1643 from strain BLS256; used to create mutant *Δ1643* | This study |
| pkMS1-*1946* | Km^R^; pKMS1 containing the left (300 bp) and right (587 bp) DNA fragment of xoc_1946 from strain BLS256; used to create mutant *Δ1946* | This study |
| pkMS1-*0582* | Km^R^; pKMS1 containing the left (211 bp) and right (567 bp) DNA fragment of xoc_0582 from strain BLS256; used to create mutant *Δ0582* | This study |
| pkMS1-*2868* | Km^R^; pKMS1 containing the left (549 bp) and right (307 bp) DNA fragment of xoc_2868 from strain BLS256; used to create mutant *Δ2868* | This study |
| pkMS1-*3249* | Km^R^; pKMS1 containing the left (523 bp) and right (271 bp) DNA fragment of xoc_3249 from strain BLS256; used to create mutant *Δ3249* | This study |
