# Supplementary material for: Transcriptome analysis of Xanthomonas oryzae pv. oryzicola exposed to H2O2 reveals horizontal gene transfer contributes to its oxidative stress response
Source: PLoS One. 2019 Oct 3;14(10):e0218844. doi: 10.1371/journal.pone.0218844 (PMC6776340; doi:10.1371/journal.pone.0218844)
Supplement: S2 Table — (DOCX) [file pone.0218844.s002.docx]

**S2 Table** Primers used in this study.

| Purpose | Primers | Sequence (5’→3’; restriction sites underscored) | Description |
| --- | --- | --- | --- |
| Mutagenesis | 1643-1F | AAA**GGATCC**GGCAACATTTCGACAACCCG | Amplifies 196 bp  fragment left of *xoc_1643* |
|  | 1643-1R | AAA**GAATTC**CGCAGCACCTCAGCAATCA |  |
|  | 1643-2F | AAA**GAATTC**GAGCCGTATTGCGCTGTTTC | Amplifies 434 bp  fragment right of *xoc_1643* |
|  | 1643-2R | AAA**CTGCAG**CCAGTTGCTGTCGCTTTGC |  |
|  | 1946-1F | AAA**GGATCC**AAGTACGCCCGCAGTTGG | Amplifies 300 bp  fragment left of *xoc_1946* |
|  | 1946-1R | AAA**GAATTC**CGGTGGAATCGGAAGGAAT |  |
|  | 1946-2F | AAA**GAATTC**TACCGCACCAAACAGGAGG | Amplifies 587 bp  fragment right of *xoc_1946* |
|  | 1946-2R | AAA**GTCGAC**TCGCTGGCGTTGTAATCG |  |
|  | 2868-1F | AAA**GGATCC**GTTTGCGATGTATGGGTGAAGG | Amplifies 549bp  fragment left of *xoc_2868* |
|  | 2868-1R | AAA**GAATTC**GCGAATGAGGCACAGAGGC |  |
|  | 2868-2F | AAA**GAATTC**TGGTGAATGGACAACTGGCTAA | Amplifies 307 bp  fragment right of *xoc_2868* |
|  | 2868-2R | AAA**CTGCAG**GTAGTGGCTCTGCACAAGACG |  |
|  | 3249-1F | AAA**GGATCC**TTTCGATCCGCGTCACCT | Amplifies 523bp  fragment left of *xoc_3249* |
|  | 3249-1R | AAA**GAATTC**TCAGGCAGGGATAGATCCACTA |  |
|  | 3249-2F | AAA**GAATTC**TATGGAACGTGGCGTGTCG | Amplifies 271 bp  fragment right of *xoc_3249* |
|  | 3249-2R | AAA**CTGCAG**TGCCTTGAATGCGTTGTCG |  |
| Complement-ation studies | 1643F | AAA**AAGCTT**GTGTGCCTGGTTGCCGCCAGT | 663 bp fragment containing intact *xoc_1643* and promoter |
|  | 1643R | AAA**GAATTC**ATTGAAGGACGTTGCATTCAT |  |
|  | 1946F | AAA**AAGCTT**GTGTTGAACAGAACTCCGCTC | 2625 bp fragment containing intact *xoc_1946* and promoter |
|  | 1946R | AAA**GAGCTC**TGGCGCAACCGACAAATGCCA |  |
|  | 2868F | AAA**AAGCTT**CATAGAGTCCATCACGTCCCC | 369 bp fragment containing intact *xoc_2868* and promoter |
|  | 2868R | AAA**GAATTC**ATGCTCGATGTGCTGCGTCAG |  |
|  | 3249F | AAA**AAGCTT**CGCGCGTTTGTGCAAGCGCAC | 357 bp fragment containing intact *xoc_3249* and promoter |
|  | 3249R | AAA**GAATTC**ATGAACCTCGCGCCGACCACG |  |
